# European Correspondence

**Published:** 1868

**Authors:** W. S. Armstrong

**Affiliations:** Paris, France


### ARTICLE III.

### EUROPEAN CORRESPONDENCE.

PARIS, FRANCE, March 1st, 1868.

*To Editors Atlanta Medical and Surgical Journal:*

Among the classes that are compelled to seek relief in hospitals, it is well known the percentage of phthisis is large. These subjects, the offspring generally of scrofulous parents, brought up in the most unfavorable and crowded portions of our cities, working in poorly ventilated shops during the day, and often compelled to subsist upon poor and insufficient food, fall easy victims to this scourge of the human race. Amidst the long and laborious researches in this field of medical science, from the infancy of medicine to the present day, it is unpleasant to have to confess that but little has been arrived at in the way of treatment—that the most we can hope for is to sustain the patient by good nutritious diet and exercise in the open air. That many indications do arise, in the march of this disease, where a timely and wise administration of drugs may not only alleviate suffering, but even prolong life, is nevertheless true. But even in this we are too often doomed to disappointment. For when digestion is bad, and the system fails to appropriate to itself an amount of aliment sufficient to supply the waste, the decline commences, and is more or less rapid in proportion as this function, the integrity of which is our only hope, suffers.

Of late years it has been a subject of remark that phthisis has greatly increased. My attention was recalled to this fact recently in looking over the report of the hospitals of this city for the year which has just closed. In it the reporter mentioned particularly the progressive increase of this malady from year to year, notwitstanding the theoretical and practical investigations which have been pursued with so much zeal. This report says that "out of 20 deaths registered in the service of M. Moissenet at the Hotel-Dieu for the month of November last, there were 15 from phthisis pulmonalis; and of 903 cases of tuberculosis, noted for all the hospitals during the months of November and December, 1867, there were 477 deaths—52.82 per cent! And this mortality is such that the number of deaths from phthisis pulmonalis is greater than the total number from all the diseases united." That this is not the result of accident—of the assemblage of an unusual number of these cases, but that it is the rule, is clearly shown by a comparison with the mortality of previous years. The percentage of deaths in 1866 was 51.47, and during the same year, while the cholera was raging in the city, its mortality amounted to only 31.77 per cent. This report further says that the "medium number of deaths which occur annually at Paris being 50,000, it can be safely said that 8,000, or one in six, die of phthisis pulmonalis."

This is a frightful record for the ravages of one disease. It calls loudly for therapeutic and hygenic measures to come to its relief. These frightful mortality tables seem already to have awakened here a new zeal in the investigations of this malady. It has been but a short time since M. M. Herard and Corneil have written a remarkable work on tuberculosis, in which, guided by the new principles of textural anatomy and pathology, they have brought into question some of the doctrines of their predecessors. Scarcely had the interest in this production reached its height before M. Veillmain presented at the Academy of Medicine a new volume on tuberculosis, in which he takes the ground that this disease, like syphilis and glanders, is specific in its origin; in other words, that inoculation is essential to its production. The etiology of tuberculosis has been at best but imperfectly understood—the most we have known being obtained from clinical information. But should the doctrine of the inoculability be established, it would give a new point of departure in future researches, and at the same time give origin to many questions, each one of which would have to be studied anew. It is due M. Veillmain to say that he has not arrived at his conclusions by accident, but that they are the result of a series of experiments on the lower animals, which, after being inoculated with tuberculous matter, presented uniformly and invariably tuberculous deposits in the lungs. This volume has created a very animated discussion in the Academy of Medicine of some two months' duration, and which is not yet terminated. It is combatted with great vigor by the majority. M. Herard, in his remarks, agreed with the author as to the inoculability of phthisis, but also asserted his belief in its hereditary and spontaneous origin. The discussion has taken a wide range as to the pathological appearances in tuberculosis, compared with those resulting from inoculation. But, from present developments, it is hardly probable that the nosological position of phthisis will be changed. The question evidently needs more study. Should his theory be entirely disproven, M. Veillmain will at least have the honor of awakening a new zeal in this direction, and of exciting the most interesting discussions in this body that has engaged it for a long time.

While the Academy of Medicine is thus busily occupied with the discussion of the etiology of phthisis pulmonalis, M. Montard Martin, candidate for the vacancy in the therapeutical section, comes forward with the result of his mode of treating this disease by arsenical preparations. From long trial and extended observations, his results lead him strongly to advocate its use in certain conditions of this disease. He deduces a number of conclusions from his researches, of which it will only be necessary to mention a few of the most important.

Its action, he says, "is much the most efficacious in phthisis, which is torpid and marches slowly, and which is unaccompanied with fever. On the other hand, when its march is rapid, and of the acute granulous type, this treatment does not appear to have a manifest influence."

He attributes a certain number of cures to this remedy, and believes they would be largely increased if the patients did not too soon think themselves cured and leave off the treatment. Again, he adds: "To be efficacious, it is necessary that the treatment should be long continued and the medicine administered in very small doses. It is never necessary to exceed two centigrammes."

In opposition to the opinions of some, his experience justifies him in affirming that arsenic is better tolerated by those who are somewhat advanced in phthisis than in those in whom the affection has just commenced.

During one of the recent meetings of the Academy of Medicine, M. Wurz announced that he has reproduced artificially, by a series of difficult and complicated chemical processes, *névrine* a crystalizable substance extracted from the nervous centres by Liebreich.

Within a short time, I have seen both M. Jarjavy and M. Gosslin operate for oblique inguinal hernia. The former, in his observations preceding the operation, took occasion to speak of his practice when called to see these cases, in which, it strikes us, there is considerable merit. According to the teaching and practice of all surgeons in strangulated hernia, the first step is to attempt to return the viscera by taxis. Should this fail, our works on surgery recommend it to be followed by chloroform, the warm bath, blood-letting, ice to the part, tobacco clysters, and, as a last measure, to resort to the knife. With this practice he takes issue. In common with surgeons generally, he in the first place tries the taxis. But just here the routine treatment is abandoned, and he brings together some facts which give strength to his position. After one *well directed* effort to reduce the hernia by taxis has failed, he says he invariably operates. And now for his reasons: In the first place, he believes the relaxing effects of these agents are overrated—that they are not superior to the relaxing effect obtained by position—approximating the knees and trunk. In the second place, their application consumes considerable time, during which the vitality of the constricted viscera may not only be compromised, but no doubt is often destroyed. While many cannot fully agree with him in regard to the feeble therapeutic effect of the remedies mentioned above, his position in regard to the delay from this routine use seems to be well taken. It is impossible for a surgeon to determine precisely how long an operation can be postponed without danger to the patient, which, if performed early, will prevent this result. Hence, he deems it a good precept to operate immediately after a *well directed effort* by the taxis has failed to reduce the hernia; and he fortifies this view by citing the favorable results of the cases of Beyer and Berard, the Elder, who adopted this rule. The former operated for hernia 36 times, and they all recovered; the latter operated 15 times, and had 13 recoveries.

Considering the gravity of this accident and the large number of unfavorable cases from it, these happy results strongly recommend this mode of practice to the favorable consideration of the profession. It may be urged, it is true, that this practice is too precipitate—that a trial of other means may do away with the necessity of an operation in some of the cases. But, on the other hand, it can be argued with just as much reason that the delay from their use may cause sloughing of the constricted part, with all its evil consequences. Hence, we are strongly persuaded the success attending an early operation should be borne in mind. M. Gosslin, in his remarks, was equally decided in favor of an early operation, only recommending the use of chloroform in addition to the taxis.

The method of treating that most troublesome of all affections—stricture of the urethra—by galvano-caustic, first brought into notice by Cinicilli, has received a new impetus in the hands of M. M. Mallez and Tripier. Their manner of application differs from that of Cinicilli, and they claim that their cures are permanent, as the resulting cicatrix is not retractive. How many plans of treatment will be invented for this trouble it would be difficult to say. That of caustics of gradual and forcible dilatution, and that of urethrotomy, etc., have each in its turn given brilliant hopes, and at first been crowned with marvellous success. Many, too, have been the essays written on these different plans. A multiplication of remedies and essays on the treatment of any disease furnishes one of the very best evidences of its intractible nature, if it does not demonstrate its incurability.

The manner of applying the galvano-caustic by Cinicilli we will briefly indicate, and afterwards that of Mallez and Tripier. The salt which composes the caustic of Cinicilli is placed in the circuit of an electric battery and brought in contact with the organic tissue which it is proposed to cauterize. The effect of its action on the tissue is regulated at pleasure by increasing or diminishing the current which sets free the acid and alcali composing the salt.

M. Tripier was the first to demonstrate the difference of the action between the two poles—the one setting free the acid, the other the alcali. He discovered that the alcaline caustic left a soft cicatrix, not contractile, and that the acid caustic gave a cicatrix resembling that from a burn, and very contractile. The negative pole, around which the alcaline caustic is attracted, he established, gave the cicatrix of the alcaline caustics, and the positive pole, which attracts the acid, that of the acid caustics. It therefore became clear that to produce the soft cicatrix it would only be necessary to employ the regative pole and dispense with the use of the salt. Its effect will be more or less rapid in proportion to the strength of the current, and, unlike many caustics, is limited to the point of contact. The electrode of Mallez and Tripier consists of a coil of metal conducted to the stricture through a bougie which protects the canal.

The surgeon being on the right of the patient, the inner part of the left thigh is covered with several disks of argaric moistened; the positive pole terminating in a piece of charcoal, is secured in the midst over the argaric to the leg. The negative pole is then introduced through the bougie, and its extremity brought in contact with the stricture. When the current is turned on this pole, the extremity of the electrode is brought in contact with the stricture and made to cauterize in front, behind, and at the sides, taking care not to allow the point to project too far through the sound, until the obstacle is overcome and the sound passes.

Thirty-one cases have been thus treated by these surgeons. One of them, going to work immediately after the operation, was attacked with fever and died. But this is in no way attributable to the treatment. The others are represented as being permanently cured. In some, the time of operation dates as far back as two years without return of stricture. It is believed that the agent has the effect of causing the absorption of the interstitial deposit around the seat of stricture. W. S. ARMSTRONG, M. D.

